# Epidemiological trends and age-period-cohort effects on cardiovascular diseases burden attributable to ambient air pollution across BRICS

**DOI:** 10.1038/s41598-024-62295-6

**Published:** 2024-05-20

**Authors:** Nawsherwan, Shahzad Ali Khan, Sumaira Mubarik, Zhang Le, Fazli Akbar, Yan Wang

**Affiliations:** 1 School of Medicine, Xiamen Cardiovascular Hospital of Xiamen University, Fujian Branch of National Clinical Research Center for Cardiovascular Diseases, Xiamen, China; 2 Department of Public Health, School of Public Health, Health Services Academy, Islamabad, Pakistan; 3 Department of Epidemiology and Biostatistics, School of Public Health, Wuhan University, Wuhan, Hubei China; 4 Guangdong Provincial Key Laboratory of Tropical Disease Research, Department of Nutrition and Food Hygiene, School of Public Health, Southern Medical University, Guangzhou, China

**Keywords:** Cardiovascular disease burden, Ambient air pollution, BRICS, Age-period-cohort analysis, Cardiology, Risk factors

## Abstract

Long-term exposure to ambient air pollution raises the risk of deaths and morbidity worldwide. From 1990 to 2019, we observed the epidemiological trends and age-period-cohort effects on the cardiovascular diseases (CVD) burden attributable to ambient air pollution across Brazil, Russia, India, China, and South Africa (BRICS). The number of CVD deaths related to ambient particulate matter (PM) pollution increased nearly fivefold in China [5.0% (95% CI 4.7, 5.2)] and India [5.7% (95% CI 5.1, 6.3)] during the study period. The age-standardized CVD deaths and disability-adjusted life years (DALYs) due to ambient PM pollution significantly increased in India and China but decreased in Brazil and Russia. Due to air pollution, the relative risk (RR) of premature CVD mortality (< 70 years) was higher in Russia [RR 12.6 (95% CI 8.7, 17.30)] and India [RR 9.2 (95% CI 7.6, 11.20)]. A higher period risk (2015–2019) for CVD deaths was found in India [RR 1.4 (95% CI 1.4, 1.4)] followed by South Africa [RR 1.3 (95% CI 1.3, 1.3)]. Across the BRICS countries, the RR of CVD mortality markedly decreased from the old birth cohort to young birth cohorts. In conclusion, China and India showed an increasing trend of CVD mortality and morbidity due to ambient PM pollution and higher risk of premature CVD deaths were observed in Russia and India.

## Introduction

Cardiovascular disease (CVD) is the leading cause of premature mortality worldwide, accounting for 18.6 million deaths in 2019^1^. The low- and middle-income countries responsible for more than three-quarters of CVD deaths^2^ and CVD-related deaths would be more than 23 million worldwide in 2030^3^. Brazil, Russia, India, China, and South Africa (BRICS), are a group of upper- and lower-middle-income countries undergoing rapid politico-economic development and comprising nearly 44% of the world population. BRICS has an immense influence on the global economy with a 32% contribution to the world’s gross domestic product^4^. BRICS has experienced a high age-standardized CVD deaths due to major cardiovascular modifiable risk factors during 1992–2017^5–8^. The huge burden of CVD among the young population and productive age groups across BRICS can adversely affect national economic development and the global economy. The aging of the population has resulted in substantial heterogeneity in a high CVD burden and age-specific CVD mortality rates across BRICS. Moreover, variation in CVD burden could be attributable to the implementation of healthcare intervention and prevention policies for air pollution across BRICS^9,10^.

Air pollution including ambient particulate matter (PM) pollution (PM_2.5_) and household air pollution (HAP) from solid fuels are considered the key environmental risk factor for premature CVD mortality^11^. Air pollution particularly ambient PM pollution (PM_2.5_) changed from the 13th leading risk factor in 1990 to the 7th in 2019 for all ages disease burden^12^. In 2019, the annual average concentration of ambient PM pollution (PM_2.5_) exceeded the world health organization (WHO) air quality guideline (10 μg/m^3^) and affects more than 90% of the global population. Moreover, the trend of exposure to ambient PM pollution (PM_2.5_) is lower in developed countries, and high in developing and middle-developed countries^12,13^. BRICS countries are facing serious threats to human health associated with air pollution particularly ambient PM pollution. In Brazil, the annual PM_2.5_ concentrations range from 2.9 to 23.6 μg/m^3^ during 2010–2018^14^. From 1990 to 2017, the estimated annual concentration of PM_2.5_ was 52.7 μg/m^3^ in China^15^. In India, half of the population was exposed to PM_2.5_ above 40 μg/m^3^ during 2001–2010^16^. Similarly, Russia and South Africa showed higher concentrations of air pollutants^17,18^. Long-term exposure to air pollution has been associated with increased risk of CVD, cancer, diabetes, respiratory infection, and loss of life expectancy across the BRICS countries^14–18^.

Several epidemiological studies showed that short-term and long-term exposure to ambient air pollution had deleterious effects on human health including respiratory diseases, lung cancer, type 2 diabetes, and cardiovascular diseases^19–22^, and caused 6.67 million deaths worldwide in 2019^23^. Moreover, 4.75 million CVD deaths are attributable to ambient air pollution across the globe in 2021^11^. Previous studies have explored the time trends in air-pollution-related disease burden at the global level^11,19,22,24^. To date, few studies observed the time trends in disease burden across the BRICS^5,25,26^. However, no study determined the epidemiological trend and period-cohort effects on the burden of CVD due to air pollution across the BRICS. Therefore, the present study used data from the GBD 2019 to estimate the epidemiological trend and period-cohort impacts on the burden of CVD attributable to air pollution across the BRICS from 1990 to 2019. The findings of this study can provide recent insights to the health policymakers for prioritizing initiatives to reduce the burden of CVD caused by air pollution across the BRICS.

## Results

### Temporal trends in CVD mortality and DALYs attributable to air pollution

Across the BRICS, the all-ages CVD deaths (1.3 million to 2.0 million) and DALYs (35.4 to 49.3 million) attributable to air pollution significantly increased by 1.5% (95% CI 1.2, 1.7) and 1.2% (95% CI 1.0, 1.4) per year during the study period, respectively. However, the age-standardized CVD mortality and DALYs decreased by −2.0% (95% CI −2.3, −1.8) and −2.0% (95% CI −2.2, −1.9) per year. In 2019, across the BRICS, more than half of the CVD deaths (1.1 million) and DALYs (26.1 million) were in China. India had a marked increase in all-ages CVD deaths [2.3% (95% CI 1.6, 2.9)] and DALYs [2.0% (95% CI 1.4, 2.5)] per year. On the other hand, Russia showed a remarkable reduction in all-ages CVD deaths [−1.8% (95% CI −3.0, −0.6)] and DALYs [−2.0% (95% CI −3.7, −0.3)] per year. Brazil had the largest decline in age-standardized CVD mortality [−4.7% (95%CI: −5.0, −4.5)] and DALYs [−4.6% (95% CI −4.9, −4.4)] per year during 1990–2019 (Table 1 and Table S1).

**Table 1:** Time trend in the burden of CVD mortality attributable to ambient air pollution for both sexes across BRICS from 1990 to 2019.

CVD	ASMR/100,000			Deaths, n × 10,000		
Air pollution	1990 (95% UI)	2019 (95% UI)	AAPC (95% CI)	1990 (95% UI)	2019 (95% UI)	AAPC (95% CI)
BRICS	73 (93, 56)	41 (49, 32)	−2.0 (−2.3, −1.8)	131 (157, 108)	200 (237, 167)	1.5 (1.2, 1.7)
Brazil	55 (71, 40)	13 (17, 9)	−4.7 (−5.0, −4.5)	4 (5, 3)	3 (4, 2)	−1.3 (−1.6, −1.1)
Russia	67 (107, 31)	28 (40, 16)	−2.9 (−4.1, −1.7)	11 (17, 5)	6 (9, 3)	−1.8 (−3.0, −0.6)
India	100 (117, 85)	69 (81, 58)	−1.2 (−2.0, −0.5)	40 (46, 34)	75 (88, 64)	2.3 (1.6, 2.9)
China	104 (121, 90)	63 (73, 53)	−1.7 (−2.0, −1.4)	74 (86, 64)	114 (133, 96)	1.5 (1.2, 1.8)
South Africa	41 (47, 34)	30 (36, 24)	−1.0 (−1.7, −0.3)	0.8 (0.9, 0.7)	1.2 (1.4, 1.1)	1.4 (0.6, 2.1)
Ambient PM pollution						
BRICS	31 (51, 15)	31 (40, 23)	0.1 (−0.3, 0.3)	44 (74, 20)	150 (184, 119)	4.2 (3.8, 4.7)
Brazil	21 (37, 9)	10 (13, 7)	−2.5 (−2.7, −2.2)	1.8 (3.1, 0.8)	2.3 (3.1, 1.6)	1.0 (0.7, 1.2)
Russia	60 (101, 26)	27 (39, 15)	−2.7 (−3.8, −1.5)	10 (16, 4)	6 (9, 3)	−1.5 (−2.7, −0.4)
India	24 (40, 11)	45 (55, 34)	2.1 (1.2, 3.1)	9 (16, 4)	49 (60, 37)	5.7 (5.1, 6.3)
China	31 (52, 15)	50 (60, 41)	1.7 (1.4, 1.9)	22 (37, 10)	91 (109, 74)	5.0 (4.7, 5.2)
South Africa	22 (28, 16)	25 (31, 20)	0.5 (−0.5, 1.4)	0.4 (0.5, 0.3)	1.1 (1.2, 0.8)	2.9 (1.9, 3.8)
HAP from solid fuels						
BRICS	41 (55, 29)	9 (14, 5)	−5.0 (−5.2, −4.8)	86 (112, 61)	50 (78, 29)	−1.8 (−2.1, −1.5)
Brazil	33 (43, 25)	3 (5, 1)	−7.8 (−8.0, −7.5)	2.7 (3.5, 2.1)	0.7 (1.3, 0.3)	−4.4 (−4.6, −4.3)
Russia	7 (14, 2)	0.8 (2, 0.2)	−6.9 (−7.6, −6.2)	1.1 (2.4, 0.4)	0.2 (0.5, 0.1)	−5.8 (−6.5, −5.1)
India	75 (98, 54)	24 (34, 15)	−3.9 (−4.7, −3.1)	30 (38, 22)	26 (37, 17)	−0.5 (−1.2, 0.2)
China	72 (93, 51)	12 (21, 6)	−5.9 (−6.2, −5.6)	52 (66, 36)	22 (38, 11)	−2.8 (−3.1, −2.4)
South Africa	18 (25, 12)	4 (7, 2)	−4.5 (−5.3, −3.6)	0.3 (0.5, 0.2)	0.1 (0.3, 0.1)	−2.3 (−3.2, −1.4)

*CVD* cardiovascular disease, *ASMR* age-standardized mortality rate, *AAPC* average annual percent change, *ambient PM pollution* ambient particulate matter pollution, *HAP from solid fuels* household air pollution from solid fuels.

### Time trend in CVD mortality and DALYs attributable to ambient PM pollution

The all-ages CVD deaths (0.4 to 1.5 million) and DALYs (11.7 to 36.6 million) attributable to ambient PM pollution increased by 3.7-fold and 3.1-fold across the BRICS, respectively. India showed the fastest increasing trend of all-ages CVD deaths [5.7% (95% CI 5.1, 6.3)] and DALYs [5.6% (95% CI 4.8, 6.3)] per year. However, among the BRICS, only Russia observed a notable reduction in all-ages CVD deaths [−1.5% (95% CI −2.7, −0.4)] and DALYs [−1.7% (95% CI −3.6, 0.1)] per year. The age-standardized CVD mortality and DALYs significantly increased in India and China but decreased in Brazil and Russia (Table 1 and Table S1).

### Time trend in CVD mortality and DALYs attributable to HAP from solid fuels

The BRICS observed a significant reduction in all-ages CVD deaths [−1.8% (95% CI −2.1, −1.5)] and DALYs [−2.1% (95% CI −2.4, −1.8)] attributable to HAP from solid fuels during the study period. All BRICS countries showed significant improvement in the all-ages and age-standardized CVD DALYs and deaths due to HAP from solid fuels. In 2019, India had the highest CVD ASMR [24 (95% UI 34, 15)] per 100,000 populations, which is double that of China and eightfold higher than Brazil (Table 1 and Table S1).

### Gender-specific trend and distribution of CVD burden attributable to air pollution

**Figure 1:**
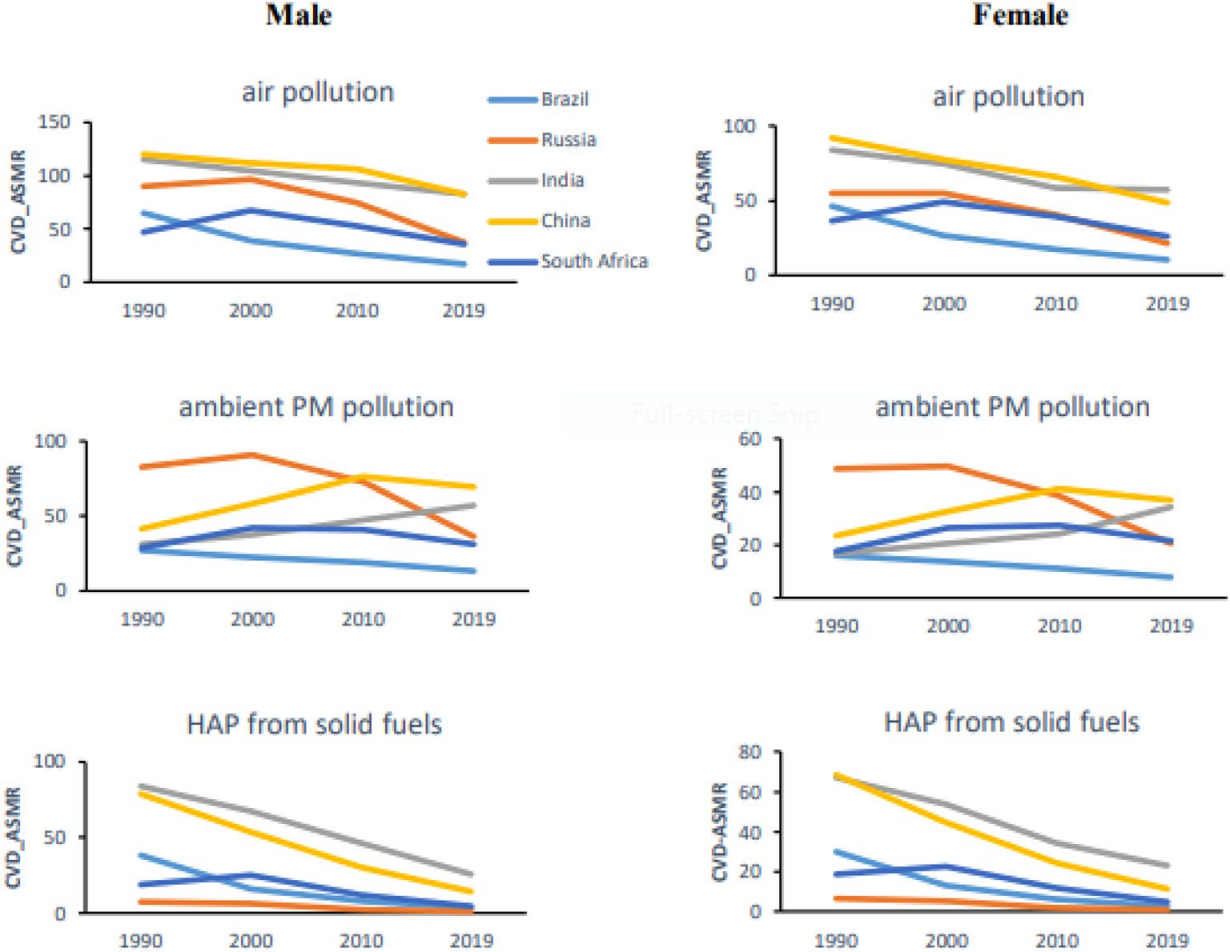
Temporal trend of age-standardized mortality rate (ASMR) of cardiovascular diseases (CVD) attributable to air pollution, ambient particulate matter (PM) pollution, and household air pollution (HAP) from solid fuels among males and females across Brazil, Russia, India, China, and South Africa from 1990 to 2019.

**Figure 2:**
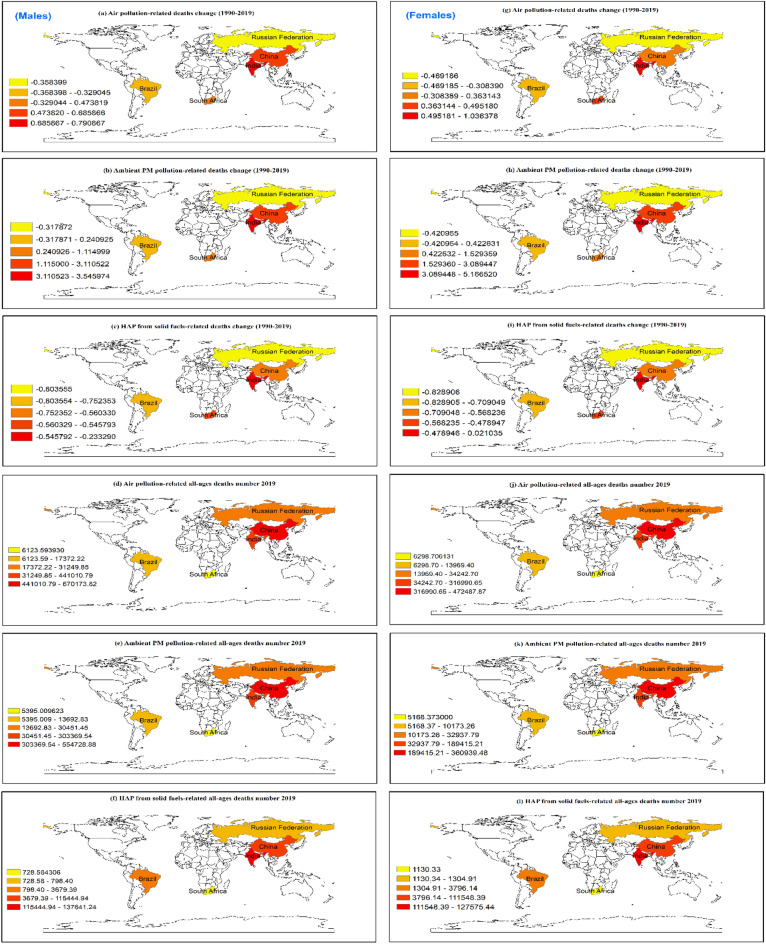
The annual rate of change in all-ages CVD deaths (1990 to 2019) and all-ages CVD death numbers in 2019 in males (left panel) and females (right panel) attributable to air pollution across BRICS countries.

**Figure 3:**
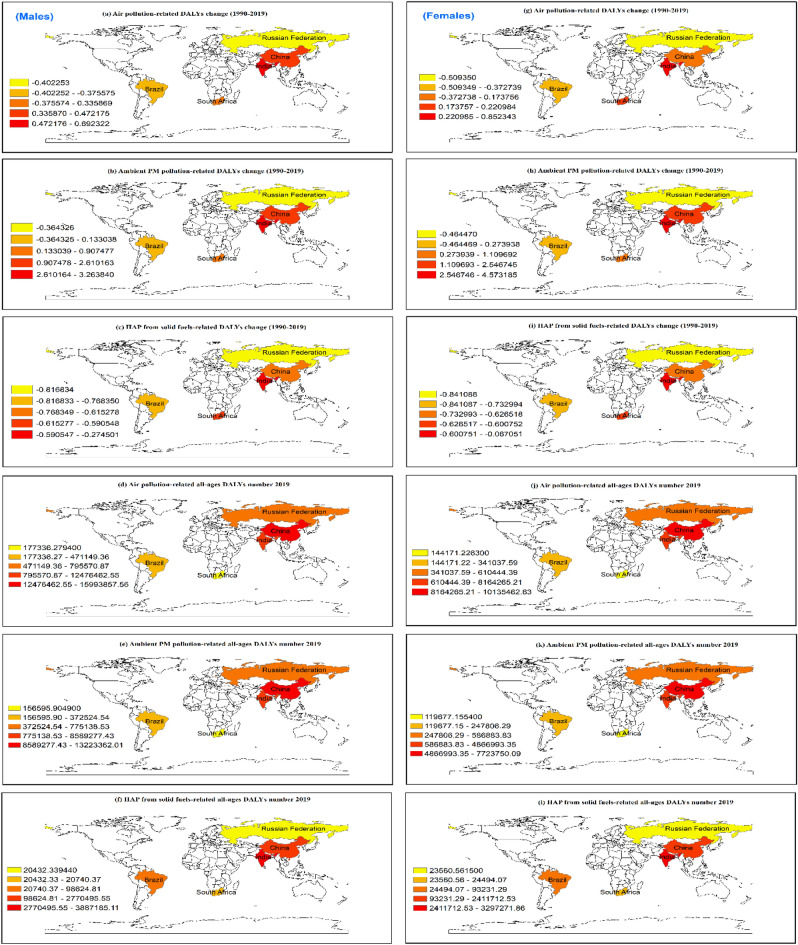
The annual rate of change in all-ages DALYs (1990 to 2019) and all-ages DALYs numbers in 2019 in males (left panel) and females (right panel) attributable to air pollution across BRICS countries.

Males had higher age-standardized CVD mortality and DALYs and showed less improvement in CVD burden attributable to air pollution, ambient PM pollution, and HAP from solid fuels than females across the BRICS. However, females in India observed a significantly upward trend in the age-standardized CVD deaths and DALYs due to ambient PM pollution than the male population. In 2019, females had higher all-ages CVD deaths due to air pollution than males in Russia and South Africa. The annual rate of change (AROC) in all-ages CVD deaths and DALYs due to ambient PM pollution was significantly higher in females than males in China and South Africa (Table 2, Figs. 1–3, Fig. S1).

**Table 2:** Time trend in the burden of age-standardized CVD mortality and DALYs rate attributable to ambient air pollution in males and females across BRICS from 1990 to 2019.

CVD ASMR	Male (AAPC (95% CI)			Female (AAPC (95% CI)		
	Air pollution	Ambient PM pollution	HAP from solid fuels	Air pollution	Ambient PM pollution	HAP from solid fuels
BRICS	−1.8 (−2.0, −1.6)	−0.1 (−0.4, 0.3)	−5.2 (−5.4, −4.9)	−2.2 (−2.4, −2.0)	−0.1 (−0.3, 0.2)	−4.9 (−5.2, −4.7)
Brazil	−4.6 (−4.8, −4.3)	−2.5 (−2.7, −2.3)	−7.9 (−8.1, −7.6)	−4.9 (−5.1, −4.6)	−2.4 (−2.7, −2.2)	−7.7 (−7.9, −7.6)
Russia	−3.0 (−4.6, −1.3)	−2.8 (−4.4, −1.2)	−6.9 (−7.8, −6.0)	−3.1 (−4.1, −2.1)	−2.8 (−3.7, −1.9)	−7.0 (−7.5, −6.4)
India	−1.1 (−1.8, −0.5)	2.0 (1.5, 2.5)	−4.0 (−4.7, −3.3)	−1.3 (−2.1, −0.5)	2.5 (1.3, 3.6)	−3.7 (−4.6, −2.8)
China	−1.3 (−1.6, 0.9)	1.8 (1.5, 2.1)	−5.8 (−6.1, −5.5)	−2.2 (−2.4, −2.0)	1.5 (1.3, 1.8)	−6.0 (−6.2, −5.7)
South Africa	−1.0 (−1.6, −0.4)	0.3 (−0.6, 1.2)	−4.9 (−5.4, −4.3)	−1.1 (−1.9, −0.2)	0.7 (−0.4, 1.8)	−4.4 (−5.4, −3.3)
CVD DALYs						
BRICS	−1.9 (−2.1, −1.7)	−0.1 (−0.5, 0.2)	−5.1 (−5.3, −4.9)	−2.3 (−2.5, −2.0)	−0.1 (−0.3, 0.2)	−5.0 (−5.2, −4.7)
Brazil	−4.5 (−4.8, −4.3)	−2.5 (−2.7, −2.3)	−7.8 (−8.0, −7.6)	−4.7 (−5.0, −4.5)	−2.3 (−2.6, −2.1)	−7.5 (−7.7, −7.4)
Russia	−2.7 (−4.5, −0.8)	−2.4 (−4.4, −0.5)	−6.7 (−7.7, −5.8)	−3.0 (−4.2, −1.9)	−2.8 (−3.9, −1.6)	−6.9 (−7.6, −6.2)
India	−1.0 (−1.6, −0.4)	2.1 (1.7, 2.6)	−3.9 (−4.5, −3.3)	−1.2 (−1.8, −0.5)	2.8 (1.8, 3.8)	−3.5 (−4.2, −2.7)
China	−1.4 (−1.7, −1.1)	1.6 (1.4, 1.9)	−5.9 (−6.1, −5.7)	−2.4 (−2.6, −2.2)	1.4 (1.1, 1.6)	−6.1 (−6.4, −5.9)
South Africa	−1.3 (−1.8, −0.8)	−0.1 (−0.9, 0.8)	−5.3 (−6.0, −4.7)	−1.7 (−2.7, −0.8)	0.1 (−0.9, 1.1)	−5.3 (−6.3, −4.3)

*CVD* cardiovascular disease, *DALYs* disability-adjusted life years, *ASMR* age-standardized mortality rate, *AAPC* average annual percent change. *ambient PM pollution* ambient particulate matter pollution, *HAP from solid fuels* household air pollution from solid fuels.

### Age-period-cohort effects on CVD mortality and DALYs attributable to air pollution

**Figure 4:**
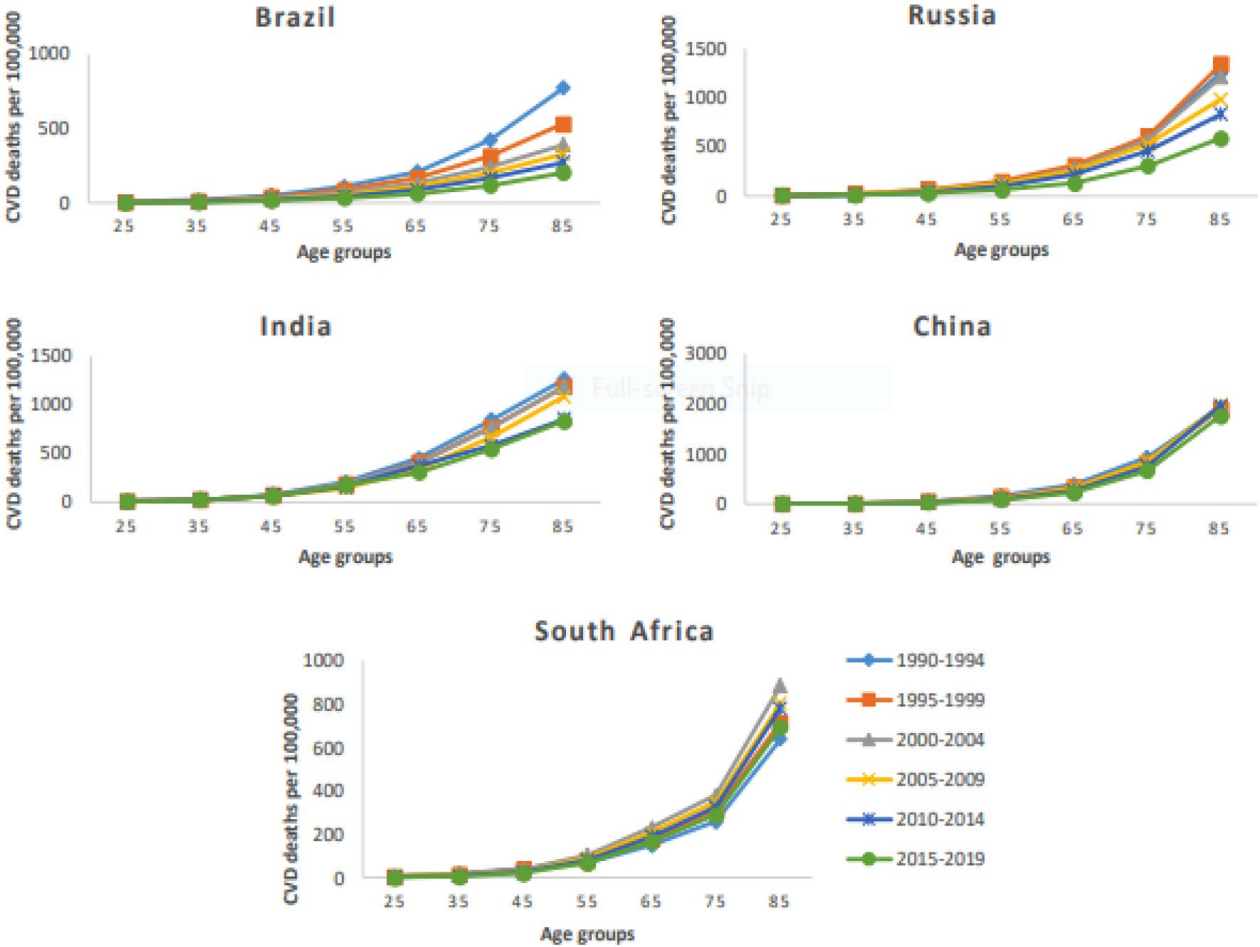
Age-specific cardiovascular diseases (CVD) mortality rate attributable to ambient air pollution by period across Brazil, Russia, India, China, and South Africa from 1990 to 2019.

**Figure 5:**
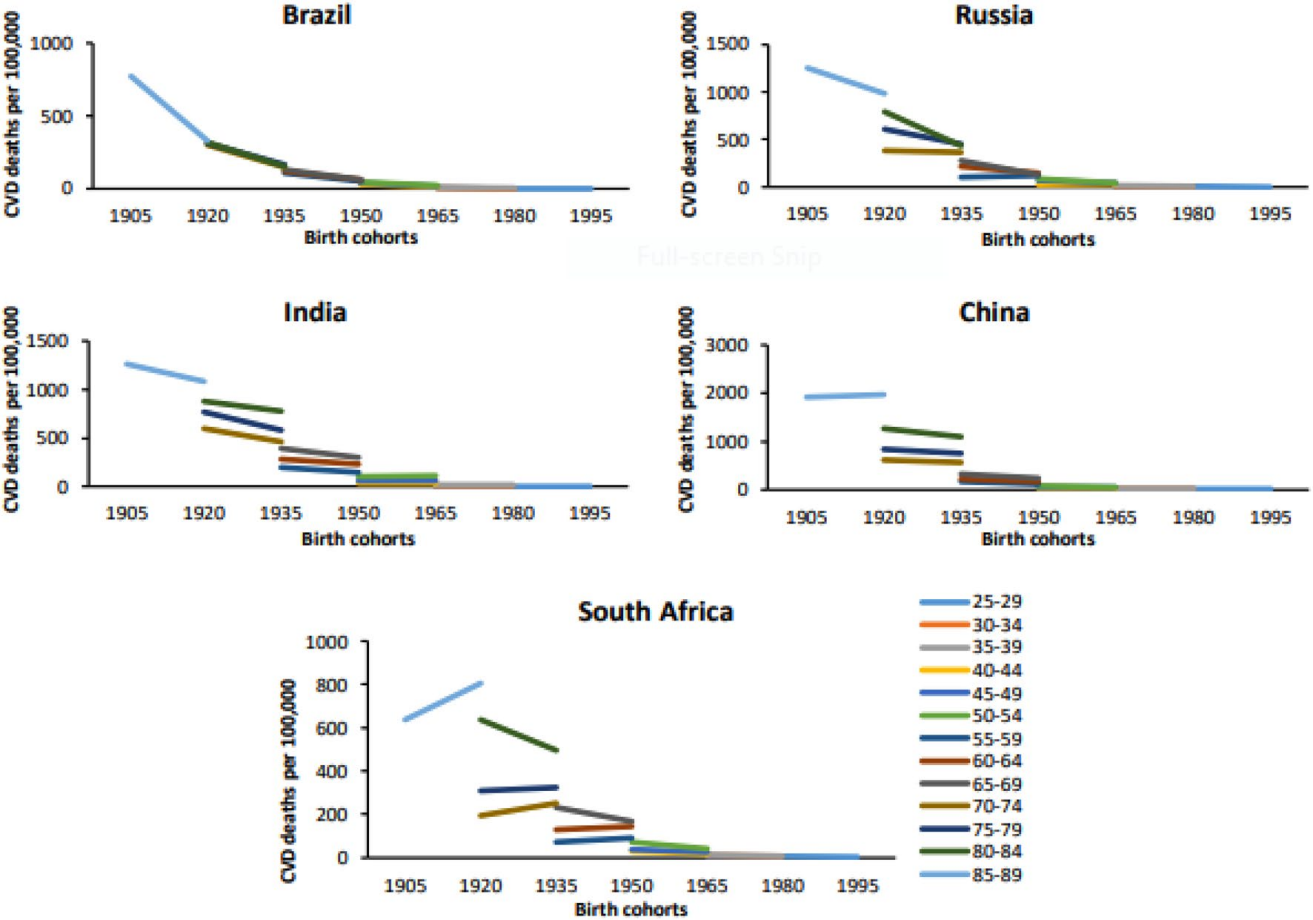
Cohort-specific cardiovascular diseases (CVD) mortality rate attributable to ambient air pollution by age groups across Brazil, Russia, India, China, and South Africa from 1990 to 2019.

The relative risk of CVD mortality and DALYs attributable to ambient air pollution exponentially increased with age across the BRICS. In the Chinese population, the relative risk of CVD mortality and DALYs spiked in the later years of life (> 80 years). The relative risk of premature CVD mortality and DALYs (< 70 years) was higher in Russia and India. However, the relative risk of premature CVD mortality and DALYs was lowest in South Africa followed by China and Brazil. The period effect showed different direction for individual country. For Brazil, the relative risk of CVD mortality and DALYs significantly decreased over the last three decades and showed the greatest improvement in the reduction of the CVD burden. Similarly, Russia showed favorable period trends of CVD burden in the last decade. In contrast, the relative risk of CVD mortality and DALYs significantly increased in India, China, and South Africa across the study period. The birth cohort effect showed a similar direction across all BRICS countries. The relative risk of CVD mortality and DALYs markedly decreased from the old birth cohort to recent birth cohorts. China had a higher relative risk of CVD burden in old birth cohorts among BRICS. For South Africa, the relative risk of CVD mortality and DALYs exponentially decreased in cohorts born after 1950 and the recent birth cohort had the lowest relative risk of CVD burden (Table 3, Table S2, Figs.4 and 5, Figs. S2 and S3).

**Table 3:** Age-Period-Cohort effects on CVD mortality attributable to ambient air pollution across BRICS.

Variables	CVD mortality (RR 95% CI)				
	Brazil	Russia	India	China	South Africa
Age					
25–29*	1.00	1.00	1.00	1.00	1.00
30–34	1.7 (2.0, 1.5)	1.9 (2.2, 1.6)	1.6 (1.7, 1.5)	1.6 (1.7, 1.4)	1.6 (1.5, 1.2)
35–39	2.9 (3.6, 2.4)	3.5 (4.2, 2.7)	2.3 (2.6, 2.1)	2.4 (2.6, 2.1)	1.7 (1.8, 1.4)
40–44	4.8 (6.1, 3.7)	5.9 (7.6, 4.3)	3.6 (4.1, 3.1)	3.8 (4.4, 3.2)	2.1 (2.5, 1.7)
45–49	7.2 (9.7, 5.4)	8.9 (11.7, 6.4)	5.9 (6.9, 5.0)	5.4 (6.3, 4.4)	3.0 (3.4, 2.3)
50–54	9.8 (13.6, 7.1)	12.5 (16.9, 8.7)	8.3 (10.7, 7.4)	7.8 (9.4, 6.2)	4.6 (5.2, 3.3)
55–59	12.7 (17.8, 8.9)	16.4 (22.7, 11.2)	12.8 (15.6, 10.5)	10.4 (12.7, 8.1)	6.3 (7.4, 4.5)
60–64	15.8 (22.9, 11.0)	22.6 (31.6, 15.2)	17.0 (21.0, 13.7)	14.1 (17.5, 10.8)	9.7 (11.5, 6.8)
65–69	20.3 (29.7, 13.7)	29.1 (41.3, 19.1)	22.0 (27.4, 17.5)	19.8 (25.0, 15.0)	12.5 (15.1, 8.7)
70–74	25.5 (37.3, 17.3)	37.7 (54.1, 24.8)	25.5 (31.9, 20.1)	30.3 (38.1, 22.7)	15.7 (19.0, 10.8)
75–79	31.2 (46.1, 21.3)	47.9 (68.8, 31.5)	30.6 (38.1, 24.3)	40.9 (52.5, 31.0)	20.0 (23.9, 13.8)
80–84	37.3 (54.1, 25.8)	59.7 (84.9, 40.0)	31.2 (38.9, 24.8)	59.8 (75.3, 45.3)	32.6 (36.7, 22.7)
85–89	41.3 (58.6, 29.1)	71.5 (99.7, 47.9)	35.5 (44.1, 28.8)	82.3 (104.7, 64.9)	44.0 (49.6, 30.0)
Period					
1990–1994*	1.00	1.00	1.00	1.00	1.00
1995–1999	0.8 (0.9, 0.8)	1.3 (1.3, 1.3)	1.1 (1.1, 1.0)	1.0 (1.1, 1.0)	1.2 (1.2, 1.2)
2000–2004	0.7 (0.8, 0.7)	1.4 (1.4, 1.3)	1.2 (1.2, 1.2)	1.0 (1.1, 1.0)	1.6 (1.6, 1.5)
2005–2009	0.7 (0.7, 0.7)	1.4 (1.4, 1.4)	1.2 (1.2, 1.2)	1.2 (1.2, 1.1)	1.4 (1.5, 1.4)
2010–2014	0.6 (0.6, 0.6)	1.3 (1.4, 1.3)	1.2 (1.2, 1.2)	1.2 (1.2, 1.2)	1.4 (1.4, 1.4)
2015–2019	0.5 (0.5, 0.5)	1.0 (1.1, 1.0)	1.4 (1.4, 1.4)	1.2 (1.2, 1.2)	1.3 (1.3, 1.3)
Cohort					
1905–1909*	1.00	1.00	1.00	1.00	1.00
1910–1914	0.8 (0.8, 0.8)	0.8 (0.9, 0.8)	0.9 (0.9, 0.9)	1.0 (1.0, 1.0)	0.9 (1.0, 0.9)
1915–1919	0.7 (0.7, 0.7)	0.7 (0.7, 0.7)	0.8 (0.8, 0.8)	1.0 (1.0, 0.9)	0.9 (0.9, 0.9)
1920–1924	0.6 (0.7, 0.6)	0.6 (0.6, 0.5)	0.7 (0.7, 0.7)	0.9 (0.9, 0.9)	0.9 (0.9, 0.8)
1925–1929	0.6 (0.6, 0.5)	0.5 (0.5, 0.5)	0.6 (0.6, 0.6)	0.9 (0.9, 0.8)	0.9 (0.9, 0.8)
1930–1934	0.5 (0.5, 0.5)	0.5 (0.5, 0.4)	0.5 (0.5, 0.5)	0.8 (0.8, 0.8)	0.9 (0.9, 0.8)
1935–1939	0.5 (0.5, 0.4)	0.4 (0.4, 0.4)	0.5 (0.5, 0.4)	0.7 (0.7, 0.7)	0.8 (0.8, 0.8)
1940–1944	0.4 (0.4, 0.4)	0.4 (0.4, 0.4)	0.4 (0.4, 0.4)	0.6 (0.6, 0.6)	0.8 (0.8, 0.8)
1945–1949	0.4 (0.4, 0.4)	0.3 (0.3, 0.3)	0.3 (0.3, 0.3)	0.5 (0.5, 0.5)	0.7 (0.7, 0.7)
1950–1954	0.3 (0.3, 0.3)	0.3 (0.3, 0.3)	0.3 (0.3, 0.3)	0.4 (0.5, 0.4)	0.7 (0.7, 0.7)
1955–1959	0.3 (0.3, 0.3)	0.3 (0.3, 0.2)	0.3 (0.3, 0.3)	0.4 (0.4, 0.3)	0.6 (0.6, 0.6)
1960–1964	0.3 (0.3, 0.3)	0.2 (0.2, 0.2)	0.3 (0.3, 0.2)	0.3 (0.3, 0.3)	0.5 (0.5, 0.5)
1965–1969	0.2 (0.3, 0.2)	0.2 (0.2, 0.2)	0.2 (0.3, 0.2)	0.3 (0.3, 0.2)	0.4 (0.5, 0.4)
1970–1974	0.2 (0.2, 0.2)	0.2 (0.2, 0.1)	0.2 (0.2, 0.2)	0.2 (0.3, 0.2)	0.3 (0.4, 0.3)
1975–1979	0.2 (0.2, 0.1)	0.2 (0.2, 0.1)	0.2 (0.2, 0.2)	0.2 (0.4, 0.2)	0.3 (0.4, 0.3)
1980–1984	0.2 (0.2, 0.1)	0.2 (0.2, 0.1)	0.2 (0.2, 0.1)	0.2 (0.2, 0.1)	0.2 (0.3, 0.2)
1985–1989	0.1 (0.3, 0.1)	0.1 (0.2, 0.1)	0.2 (0.2, 0.1)	0.2 (0.2, 0.1)	0.1 (0.2, 0.1)
1990–1994	0.1 (0.7, 0.1)	0.1 (0.4, 0.1)	0.1 (0.2, 0.1)	0.1 (0.4, 0.1)	0.1 (0.3, 0.1)
AIC	6.5	7.5	8.2	7.5	7.1
BIC	−189.6	−162.1	−136.8	−184.1	−172.1

*CVD* cardiovascular disease, *RR* relative risk, *AIC* akaike information criterion, *BIC* bayesian information criterion.

## Discussion

Our study provides a detailed analysis of GBD 2019 data to estimate the recent time trend and age-period-cohort effects on CVD burden due to ambient air pollution across the BRICS. From 1990 to 2019, the all-ages CVD deaths and DALYs attributable to air pollution and ambient PM pollution significantly increased but decreased due to HAP from solid fuels across the BRICS. At the country level, the age-standardized CVD mortality and DALYs attributable to ambient PM pollution significantly increased in India and China but decreased in Brazil and Russia. The relative risk of premature CVD mortality and DALYs (< 70 years) was higher in Russia and India. Moreover, China had a higher relative risk of CVD burden in the old age group and early birth cohorts.

### Temporal trends of cardiovascular diseases burden attributable to ambient air pollution

We observed notable differences in both rates and time trends within the BRICS countries. Brazil had a ~ fivefold lowest age-standardized rate of CVD burden attributable to air pollution compared with India in 2019. Moreover, Brazil showed the largest reduction in CVD burden due to air pollution and HAP from solid fuels during the study period. Over the last few decades, the Brazil government has successfully implemented programs to improve the air quality by reducing the emission of primary pollutants by stationary and mobile sources^27^. As a result of Brazil’s emission control policies on air quality, the air pollutants significantly reduced during 1996–2009^28^. Primary healthcare reforms such as the Family Health Program and integrated care for the management of non-communicable diseases^29^, and the investment in air pollution control^28^ are likely to have contributed to the remarkable reduction of the CVD burden attributable to air pollution in Brazil.

Russia standout for its significant reduction in all-ages and age-standardized CVD burden attributable to all categories of air pollution particularly ambient PM pollution. In 1990, the WHO European centre for environment and health initiated the ‘air quality and health’ program for Europe to assess the impact of air pollution on human health^30^. During 1994–2004, the WHO conducted several quality assurance and control programs for air quality management and air pollution control to monitor air pollution impact on population health in the European Region including Russia^30^. In 2005, the WHO carried out a workshop in Eastern Europe, Caucasus, and Central Asia (EECCA), to highlight the current and future strategies for the prevention of the health impact of air pollution in Russia and other EECCA countries^31^. Country-level programs and workshops for air pollution control, strong government commitments, and public health policies could be major contributors in the distinguishing reduction of the CVD burden attributable to ambient air pollution in Russia.

China had the highest age-standardized CVD mortality rate due to ambient PM pollution followed by India in 2019. Both countries observed remarkably increasing trends in the age-standardized CVD mortality and DALYs attributable to ambient PM pollution. Ambient PM_2.5_ is among the top four risk factors contributing to deaths and DALYs in China^32^, and half of the population was exposed to PM_2.5_ above 40 μg/m^3^ in India^16^. China and India with large population sizes, owning significant rural areas that are economically disadvantaged, rapidly developing industries and unbalanced public health caused disproportionately high disease burdens attributable to ambient PM pollution. In China (2000–2010) and India (2013), the health care cost due to air pollution reached up to 6.5 and 8.5% of annual gross domestic product (GDP), respectively^24^. The Chinese and Indian governments launched several air pollution prevention and control action plans such as controlling power plants and bringing down levels of PM_2.5_ and PM_10_ by restricting vehicle emissions to improve air quality^33–35^. Hopefully, these government level strong commitment and serious action could reduce the CVD burden and health care expenditure due to ambient PM pollution.

South Africa showed a significantly increasing trend in all-ages CVD deaths and the slowest reduction in age-standardized CVD mortality due to air pollution. A recently published report on pollution and health observed that pollution remains a major threat to health and prosperity in low-income and middle-income countries (LMICs)^36^. In Africa, due to rapid industrialization, urbanization, and building infrastructure, ambient air pollution is still the predominant cause of pollution-related diseases and deaths. As a result of increased ambient air pollution, the number of non-communicable disease (NCD) deaths due to air pollution has begun to increase in many African countries^37,38^. Previous studies observed that air pollution exceeded the daily WHO guidelines and found a robust association between CVD mortality and disability in South Africa^39,40^.

### Age-period-cohort effects on CVD burden attributable to ambient air pollution

The risk of CVD burden attributable to ambient air pollution significantly increased with age across the BRICS countries. The risk ratio of CVD mortality and DALYs spiked in the later years of life (> 80 years) in the Chinese population. However, the relative risk of premature CVD mortality and DALYs (< 70 years) was higher in Russia and India. It is evident that age is an independent risk factor for CVD and its incidence increases with aging regardless of gender^41,42^. In China, the highest CVD burden in the later years of life could be attributed to its aging population^5^. In 2005, India and Russia were the second and fifth leading countries with premature CVD mortality (≥ 30 years) due to PM_2.5_ and ozone (O_3_)^43^_._ Russia had the highest CVD burden in young adults 20 to 39 years of age and India showed the highest CVD mortality among adults aged 35 to 60 years^5,44^. Premature CVD mortality due to particulate air pollution causes a huge economic loss in both countries and it is indispensable for public health planners and policymakers to take serious action at the local and national level to mitigate the complex problem of air pollution^45,46^.

Our findings show a consistently increasing period effect on CVD mortality attributable to air pollution in India. It reflects that India failed to overcome the air pollution challenge over the last three decades. At the global level, India is the major contributor to ambient air pollution and more than 90% of the population is exposed to a higher concentration of ambient PM pollution (> 10 μg/m^3^) according to the WHO air quality guidelines^47^. Rapid industrial and vehicle emissions and the high use of biomass fuels such as wood, dung, or coal caused poor air quality in both urban and rural India^48^. Besides atmospheric transport and anthropogenic emissions, poor access to CVD treatment, poor diet, poor lipid profile, increasing prevalence of hypertension, and high fasting plasma glucose country-wide may contribute to increasing risk of CVD burden over the study period^5^.

The relative risk of the birth cohort effect on the burden of CVD due to air pollution showed a monotonically declining pattern across the BRICS when compared to the reference birth cohort. Furthermore, the risk ratio of CVD mortality in China due to air pollution was larger for early birth cohorts and lower for recent birth cohorts. In China, exposure to HAP from solid fuels has shown a declining trend and a reduction in PM_2.5_ concentrations has been observed in recent years^49^. The Chinese government has prioritized combating ambient PM_2.5_ pollution, and during the past few years, several air pollution control measures have been implemented^49,50^. The lower risk ratio of CVD burden in the young birth cohorts in China could be attributed to improvement in healthcare facilities, healthcare coverage, public health initiatives in CVD prevention, and high socioeconomic status^5,6^.

Over the last 30 years, Brazil and Russia have achieved significant improvements in the CVD mortality and DALYs attributable to air pollution. However, still Russia and India experienced a higher risk of premature mortality (< 70 years) due to air pollution among the BRICS countries. Moreover, India, China, and South Africa showed a significant increasing trend in CVD burden attributable to ambient PM pollution which poses a significant public health challenge for the aforementioned countries. HAP from solid fuels is also a major risk of the highest CVD ASMR in India. These findings suggest that BRICS countries should prioritize their challenges according to each country’s national conditions and air pollution status. Brazil can serve as a reference for Russia, India, China, and South Africa in the remarkable reduction of the CVD burden by reducing the emission of primary pollutants by stationary and mobile sources^28^ and strengthening the primary healthcare system^29^. Renewable energy and biomass energy consumption could control environmental pollution^51,52^ which may help to reduce the CVD burden attributable to air pollution across the BRICS. Moreover, state-of-the-art technologies such as image-based air quality detection methods, deep learning and image-based model, and swin transformer should be adopted at governmental level across all BRICS countries to timely monitor and estimate the air quality in urban areas^53,54^. Besides controlling environmental pollution, adequate access to healthcare services for CVD diagnosis and treatment, a healthy diet, and physical activity may reduce the overall CVD mortality across the BRICS and premature CVD mortality, particularly in Russia and India^5^.

### Limitations of the study

Our study has several important limitations. First, our analysis is based on GBD 2019 and all GBD limitations are applicable to our findings as reported previously^55^. Second, data for CVD due to ambient ozone pollution are unavailable in GBD 2019 and could not find its burden across BRICS. Third, the age-period-cohort effect on CVD burden attributable to ambient PM pollution and HAP from solid fuels was not conducted across the BRICS. Fourth, we used the age-period-cohort model with the intrinsic estimator which has certain limitations such as assuming certainty in uncertain data^56^. Fifth, we only estimated the temporal trend and age-period-cohort effect for the overall CVD burden attributable to air pollution but not for the specific CVD sub-categories which might bias our findings.

### Conclusions and implications

In conclusion, India had the largest CVD ASMR attributable to HAP from solid fuels and China had the largest CVD ASMR attributable to ambient PM pollution in 2019. The age-standardized CVD burden attributable to ambient PM pollution significantly increased in India and China but decreased in Brazil and Russia during the study period. Russia and India experienced a higher relative risk of premature CVD deaths and DALYs (< 70 years) due to ambient air pollution. Moreover, China had a higher relative risk of CVD burden in the old age group and early birth cohorts. These findings can be used by the health policymakers to prioritize strategies and invest in the control of air pollution particularly in ambient PM pollution to reduce its major adverse effect on population health, especially in India and China.

## Materials and methods

### Data source

Between 1990 to 2019, the data was extracted from the free online database of global burden of diseases (GBD) study 2019 (GBD 2019, http://ghdx.healthdata.org/ gbd-results-tool)^57^ (accessed on January11, 2023). Moreover, data on CVD burden including number of deaths, age-specific deaths rate, age-standardized deaths and disability-adjusted life years (DALYs) rates due to ambient air pollution were extracted. GBD estimates a number of disease and injury indicators at local, national, and global levels, including DALYs, years lived with disability (YLDs), years of life lost (YLLs), prevalence, incidence, and death rate. In order to conduct various studies, GBD acquired data in collaboration with the world health organization (WHO), global health observatory, and world bank open data. the institute for health metrics and evaluation (IHME), University of Washington, is in charge of collecting and managing the GBD data. Therefore, the University of Washington institutional review board examined and approved a waiver of informed consent^58,59^.

### Variables of interest

In our study, the observed independent risk factors were air pollution, including ambient PM pollution (PM_2.5_) (defined as the annual gridded concentration of PM_2.5_) and HAP from solid fuels (defined as the percentage of households using solid cooking fuels and the corresponding exposure to PM_2.5_)^49^. Moreover, CVD data including number of deaths, age-specific deaths rate, age-standardized deaths and DALYs rates across the BRICS counties were the outcome variables. DALYs are the sum of YLLs and YLDs^59^.

### Statistical analysis

#### Temporal trend analysis (1990–2019)

Joinpoint regression analysis was used to estimate the average annual percentage change (AAPC) for CVD and DALYs from 1990 to 2019. AAPC indicates the changes in CVD burden in the whole study period (1990–2019). This model divides the temporal trend into several statistically significant temporal trends called the annual percentage change (APC) which indicates the burden of CVD in each small period identified by the joinpoint regression model. The temporal trend is considered positive when the APPC or APC > 0 with its 95% confidence interval (CI), however, the APPC or APC < 0 with its 95% CI shows a negative trend. Moreover, the trend is considered stable when there is no positive or negative trend. We determined AAPCs and their 95% CIs of CVD burden both males and females. Moreover, we evaluated the geographical changes and trends in CVD burden in males and females using a graphical representation. The annual rate of change (AROC) was calculated by using the formula [i.e., 100 × ln (2019 rate/1990 rate) to measure trends over the last 30 years. A positive AROC shows an increasing trend while a negative AROC indicates a declining trend in CVD burden. AROC and AAPC are considered significant when P < 0.05. The joinpoint regression software (version 4.9.1.0 (April 2022) was used for data analysis provided by the surveillance research program of the U.S. national cancer institute (NCI).

#### Age-period-cohort (APC) analysis

To estimate the independent effects of age, period, and cohort on the burden of CVD related to air pollution, we employed an age-period-cohort (APC) model. The relationship between the burden of CVD and different age groups is known as the “age effect.” The term “period effect” refers to influencing factors, such as historical occurrences and environmental circumstances, that have an immediate impact on people of all ages. It depicts fluctuation in the CVD burden across time. The cohort effect reflects changes in various lifestyles and variations in the burden of CVD among the same year of birth cohorts^60^. With the APC analysis, collinearity (birth cohort = period − age) is a prevalent problem. The three independent linear APC variables—age, period, and cohort—cannot be identified since the linearity of the two variables affects the APC model. By generating a distinct set of trend estimates independent of any arbitrary assignment of identifying limitations on age, period, or cohort coefficients that may not be verified in the data itself, the APC model with the intrinsic estimator (IE) was utilized to solve the collinearity problem^61^. The APC analysis using the IE approach estimates coefficients for the age, period, and cohort effects. These coefficients were used to construct the exponential value [exp(coef.) = ecoef.], which represents the risk ratio (RR) of a specific age, period, or birth cohort in comparison to the reference group. Age-specific CVD rates were appropriately divided into 13 age groups (ranging from 25–29 years old to 85–89 years old) in the APC model using the IE approach. It consists of 18 birth cohorts (period-age) (from 1905–1909 to 1990–1994) and 6 periods with 5 year intervals (from 1990–1994 to 2015–2019). Y = log (M) = μ + αage1 + βperiod1 + γcohort1 + ε is the general form of the APC model. Here, M represents the incidence rate in the age groups, α, β, and γ denote the functions of age, period, and cohort effect, while μ, and ε stand for the intercept item and random error. The APC model was used to decompose the three trends and estimate efficient results^62^. Additionally, the degree of model fitting was estimated and examined using the akaike information criterion (AIC) and bayesian information criterion (BIC). For the APC analysis, we used the Stata 15.0 program (College Station, Texas, USA).

### Ethics approval and to participate

Ethical review and approval was not required for the study on human participants in accordance with the local legislation and institutional requirements. Written informed consent for participation was not required for this study in accordance with the national legislation and the institutional requirements.

### Supplementary Information

Supplementary Information.

## Data Availability

The original contributions presented in the study are included in the article/Supplementary material, further inquiries can be directed to the corresponding author.
