# Supplementary material for: Epidemiological trends and age-period-cohort effects on cardiovascular diseases burden attributable to ambient air pollution across BRICS
Source: Sci Rep. 2024 May 20;14:11464. doi: 10.1038/s41598-024-62295-6 (PMC11106240; doi:10.1038/s41598-024-62295-6)
Supplement: Supplementary file 1 — Supplementary Information. [file 41598_2024_62295_MOESM1_ESM.pdf]

Table S1: Time trend in the burden of CVD DALYs attributable to ambient air pollution for both sexes across BRICS from 1990 to 2019.

**CVD**	**Age-standardized DALYs /100,000**			**DALYs, n×10,000**		
**Air pollution**	1990 (95%UI)	2019 (95%UI)	AAPC (95%CI)	1990 (95%UI)	2019 (95%UI)	AAPC (95%CI)
BRICS	1699 (2114, 1317)	937 (1138, 751)	-2.0 (-2.2, -1.9)	3540 (4192, 2954)	4930 (5817, 4134)	1.2 (1.0, 1.4)
Brazil	1333 (1717, 972)	335 (436, 245)	-4.6 (-4.9, -4.4)	129 (165, 94)	81 (105, 59)	-1.6 (-1.8, -1.4)
Russia	1452 (2269, 683	621 (893, 359)	-2.8 (-4.6, -1.0)	257 (402, 120)	140 (201, 80)	-2.0 (-3.7, -0.3)
India	2365 (2730, 2058)	1709 (1999, 1454)	-1.1 (-1.7, -0.5)	1177 (1341, 1029)	2064 (2419, 1754)	2.0 (1.4, 2.5)
China	2274 (2625, 1970)	1328 (1546, 1130)	-1.8 (-2.1, -1.5)	1949 (2253, 1687)	2612 (3053, 2213)	1.0 (0.7, 1.3)
South Africa	1071 (1232, 905)	692 (818, 569)	-1.4 (-2.1, -0.6)	25 (28, 21)	32 (38, 26)	0.9 (0.1, 0.7)
**Ambient PM pollution**						
BRICS	736 (1186, 372)	726 (912, 544)	-0.1 (-0.3, 0.3)	1170 (1960, 548)	3666 (4465, 2872)	4.0 (3.5, 4.5)
Brazil	531 (929, 242)	256 (336, 181)	-2.4 (-2.6, -2.2)	52 (91, 23)	62 (81, 43)	0.6 (0.4, 0.8)
Russia	1305 (2143, 567)	602 (868, 344)	-2.5 (-4.4, -0.7)	231 (379, 101)	136 (195, 77)	-1.7 (-3.6, 0.1)
India	575 (967, 280)	1113 (1368, 857)	2.4 (1.7, 3.2)	288 (488, 138)	1345 (1652, 1029)	5.6 (4.8, 6.3)
China	681 (1146, 323)	1066 (1268, 866)	1.5 (1.3, 1.8)	584 (984, 273)	2094 (2502, 1699)	4.5 (4.3, 4.7)
South Africa	591 (747, 447)	592 (720, 473)	-0.1 (-0.9, 0.9)	13 (17, 10)	27 (33, 22)	2.3 (1.5, 3.2)
**HAP from solid fuels**						

BRICS	962 (1258, 686)	211 (327, 123)	-5.0 (-5.3, -4.8)	2369 (3036, 1703)	1264 (1940, 747)	-2.1 (-2.4, -1.8)
Brazil	802 (1027, 600)	79 (141, 36)	-7.7 (-7.8, -7.5)	77 (98, 57)	19 (34, 8)	-4.7 (-4.8, -4.6)
Russia	146 (308, 56)	19 (52, 4)	-6.8 (-7.7, -5.8)	25 (54, 9)	4 (12, 1)	-5.9 (-6.9, -5.0)
India	1790 (2279, 1321)	596 (833, 390)	-3.7 (-4.4, -3.0)	889 (1123, 661)	718 (1004, 470)	-0.7 (-1.4, 0.1)
China	1593 (2023, 1122)	262 (447, 133)	-6.0 (-6.3, -5.7)	1365 (1744, 966)	518 (881, 264)	-3.3 (-3.6, -2.9)
South Africa	480 (654, 330)	99 (163, 49)	-5.2 (-6.0, -4.4)	11 (15, 7)	4 (7, 2)	-3.1 (-4.2, -2.1)

Note: CVD, cardiovascular disease; DALYs, disability-adjusted life years; AAPC, average annual percent change. ambient PM pollution, ambient particulate matter pollution; HAP from solid fuels, household air pollution from solid fuels.

Table S2: Age-Period-Cohort effects on CVD DALYs attributable to ambient air pollution across BRICS.

Variables	CVD DALYs (RR 95% CI)				
	Brazil	Russia	India	China	South Africa
Age					
25-29*	1.00	1.00	1.00	1.00	1.00
30-34	1.6 (1.7, 1.6)	1.8 (1.9, 1.8)	1.5 (1.5, 1.5)	1.5 (1.5, 1.4)	1.4 (1.4, 1.3)
35-39	2.6 (2.6, 2.5)	3.1 (3.1, 3.0)	2.1 (2.1, 2.1)	2.1 (2.1, 2.0)	1.5 (1.6, 1.5)
40-44	3.9 (4.0, 3.8)	4.9 (5.0, 4.8)	3.0 (3.0, 3.0)	3.1 (3.2, 3.0)	1.8 (1.8, 1.8)
45-49	5.4 (5.6, 5.3)	6.9 (7.1, 6.7)	4.5 (4.6, 4.5)	4.1 (4.2, 3.9)	2.3 (2.4, 2.3)
50-54	6.9 (7.1, 6.6)	9.0 (9.3, 8.7)	6.3 (6.4, 6.2)	5.4 (5.6, 5.3)	3.2 (3.3, 3.1)
55-59	8.1 (8.4, 7.8)	10.9 (11.2, 10.5)	8.2 (8.4, 8.0)	6.6 (6.8, 6.4)	4.0 (4.2, 3.9)
60-64	9.1 (9.4, 8.7)	13.4 (13.8, 12.8)	9.7 (9.9, 9.5)	8.1 (8.2, 7.7)	5.5 (5.7, 5.4)
65-69	10.1 (10.5, 9.7)	15.0 (15.5, 14.4)	10.9 (11.1, 10.7)	9.9 (10.1, 9.5)	6.2 (6.5, 6.1)
70-74	10.8 (11.3, 10.4)	16.4 (17.0, 15.6)	10.8 (11.0, 10.5)	12.7 (13.1, 12.2)	6.6 (6.9, 6.5)
75-79	11.0 (11.4, 10.5)	16.9 (17.6, 16.3)	10.7 (10.9, 10.4)	14.3 (14.7, 13.6)	6.9 (7.2, 6.8)
80-84	10.4 (10.8, 10.0)	16.7 (17.3, 16.0)	8.7 (8.9, 8.5)	16.3 (16.8, 15.6)	8.9 (9.3, 8.7)
85-89	9.0 (9.4, 8.7)	15.4 (16.0, 14.9)	7.9 (8.0, 7.7)	17.8 (18.4, 17.1)	9.5 (9.8, 9.2)
Period					
1990-1994*	1.00	1.00	1.00	1.00	1.00
1995-1999	0.8 (0.8, 0.8)	1.3 (1.3, 1.3)	1.0 (1.0, 1.0)	0.9 (0.9, 0.9)	1.1 (1.1, 1.1)
2000-2004	0.7 (0.7, 0.7)	1.3 (1.3, 1.3)	1.1 (1.1, 1.1)	1.0 (1.0, 1.0)	1.4 (1.4, 1.4)
2005-2009	0.6 (0.6, 0.6)	1.3 (1.3, 1.3)	1.0 (1.0, 1.0)	1.0 (1.1, 1.0)	1.2 (1.2, 1.2)
2010-2014	0.5 (0.5, 0.5)	1.2 (1.2, 1.2)	1.1 (1.1, 1.1)	1.0 (1.0, 1.0)	1.2 (1.2, 1.2)
2015-2019	0.4 (0.4, 0.4)	0.8 (0.8, 0.8)	1.1 (1.1, 1.1)	1.0 (1.0, 1.0)	1.1 (1.1, 1.1)
Cohort					
1905-1909*	1.00	1.00	1.00	1.00	1.00
1910-1914	0.9 (0.9, 0.9)	0.9 (0.9, 0.9)	0.9 (0.9, 0.9)	1.0 (1.0, 1.0)	1.0 (1.0, 1.0)
1915-1919	0.8 (0.8, 0.8)	0.7 (0.7, 0.7)	0.8 (0.9, 0.8)	1.0 (1.0, 1.0)	1.0 (1.0, 1.0)
1920-1924	0.7 (0.7, 0.7)	0.6 (0.6, 0.6)	0.8 (0.8, 0.8)	1.0 (1.0, 1.0)	1.0 (1.0, 1.0)
1925-1929	0.7 (0.7, 0.7)	0.5 (0.6, 0.5)	0.7 (0.7, 0.7)	1.0 (1.0, 1.0)	1.0 (1.0, 1.0)
1930-1934	0.6 (0.6, 0.6)	0.5 (0.5, 0.5)	0.6 (0.6, 0.6)	0.9 (0.9, 0.9)	1.0 (1.0, 1.0)
1935-1939	0.6 (0.6, 0.6)	0.5 (0.5, 0.5)	0.6 (0.6, 0.6)	0.8 (0.8, 0.8)	1.0 (1.0, 1.0)

1940-1944	0.5 (0.5, 0.5)	0.4 (0.4, 0.4)	0.5 (0.5, 0.5)	0.7 (0.8, 0.8)	1.0 (1.0, 1.0)
1945-1949	0.5 (0.5, 0.5)	0.4 (0.4, 0.4)	0.5 (0.5, 0.5)	0.7 (0.7, 0.7)	1.0 (1.0, 1.0)
1950-1954	0.5 (0.5, 0.5)	0.4 (0.4, 0.4)	0.4 (0.4, 0.4)	0.6 (0.6, 0.6)	1.0 (1.0, 1.0)
1955-1959	0.4 (0.4, 0.4)	0.4 (0.4, 0.4)	0.4 (0.4, 0.4)	0.5 (0.5, 0.5)	0.9 (0.9, 0.9)
1960-1964	0.4 (0.4, 0.4)	0.3 (0.3, 0.3)	0.4 (0.4, 0.4)	0.5 (0.5, 0.5)	0.8 (0.8, 0.8)
1965-1969	0.4 (0.4, 0.4)	0.3 (0.3, 0.3)	0.4 (0.4, 0.4)	0.4 (0.4, 0.4)	0.6 (0.6, 0.6)
1970-1974	0.3 (0.3, 0.3)	0.3 (0.3, 0.3)	0.4 (0.4, 0.4)	0.4 (0.4, 0.4)	0.5 (0.5, 0.5)
1975-1979	0.3 (0.3, 0.3)	0.3 (0.3, 0.3)	0.4 (0.4, 0.4)	0.3 (0.3, 0.3)	0.5 (0.5, 0.5)
1980-1984	0.3 (0.3, 0.3)	0.3 (0.3, 0.3)	0.3 (0.3, 0.3)	0.3 (0.3, 0.3)	0.4 (0.4, 0.4)
1985-1989	0.3 (0.3, 0.3)	0.2 (0.2, 0.2)	0.3 (0.3, 0.3)	0.3 (0.3, 0.3)	0.2 (0.2, 0.2)
1990-1994	0.3 (0.3, 0.3)	0.2 (0.2, 0.2)	0.2 (0.2, 0.2)	0.3 (0.3, 0.3)	0.2 (0.2, 0.2)
AIC	10.3	20.6	23.6	13.8	19.2
BIC	-158.7	598.4	797.2	41.9	500.1

Note: CVD, cardiovascular disease; DALYs, disability-adjusted life years; RR, relative risk; AIC, Akaike information criterion; BIC, Bayesian information criterion.

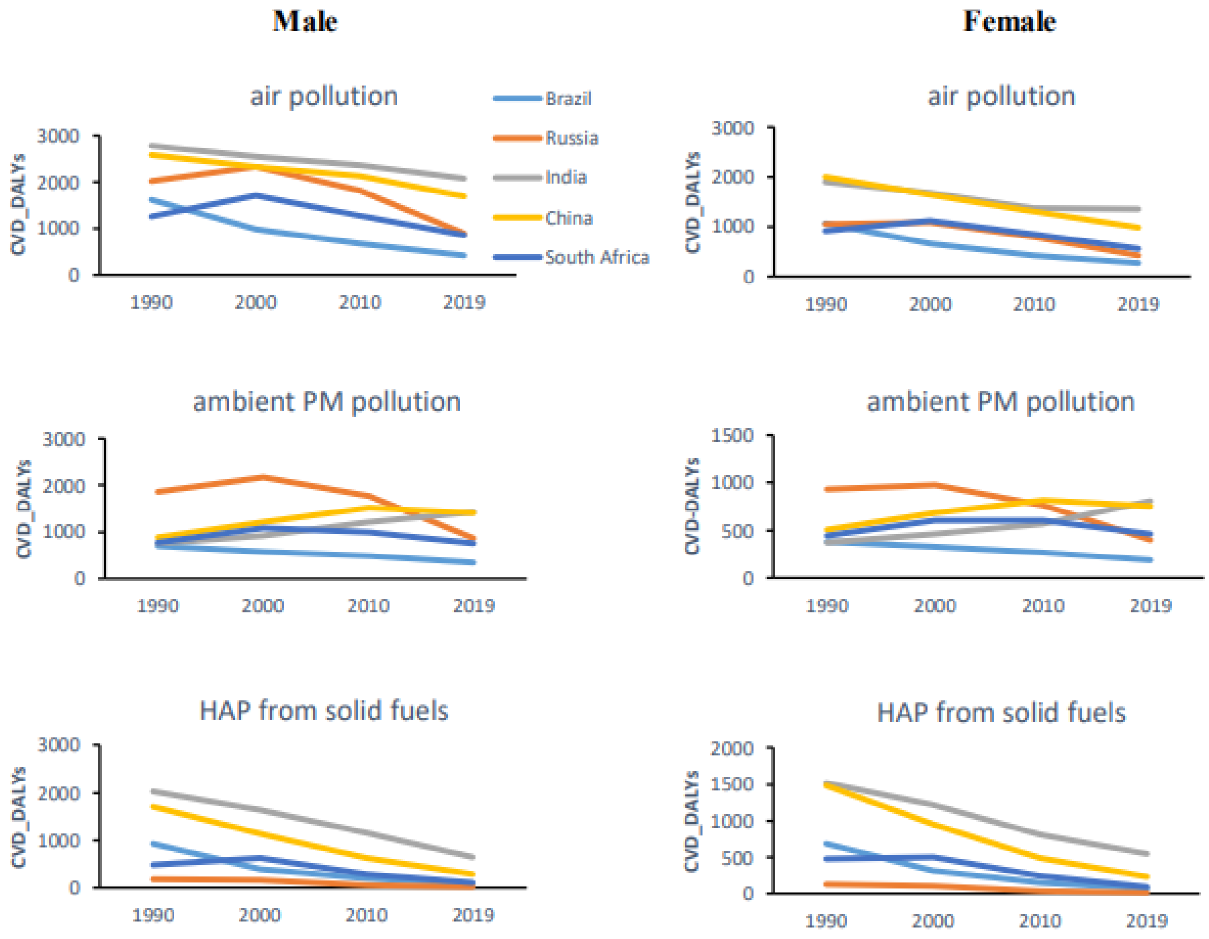

Figure S1: Temporal trend of age-standardized disability-adjusted life years (DALYs) rate due to cardiovascular diseases (CVD) attributable to air pollution, ambient particulate matter (PM) pollution, and household air pollution (HAP) from solid fuels among males and females across Brazil, Russia, India, China, and South Africa from 1990 to 2019.

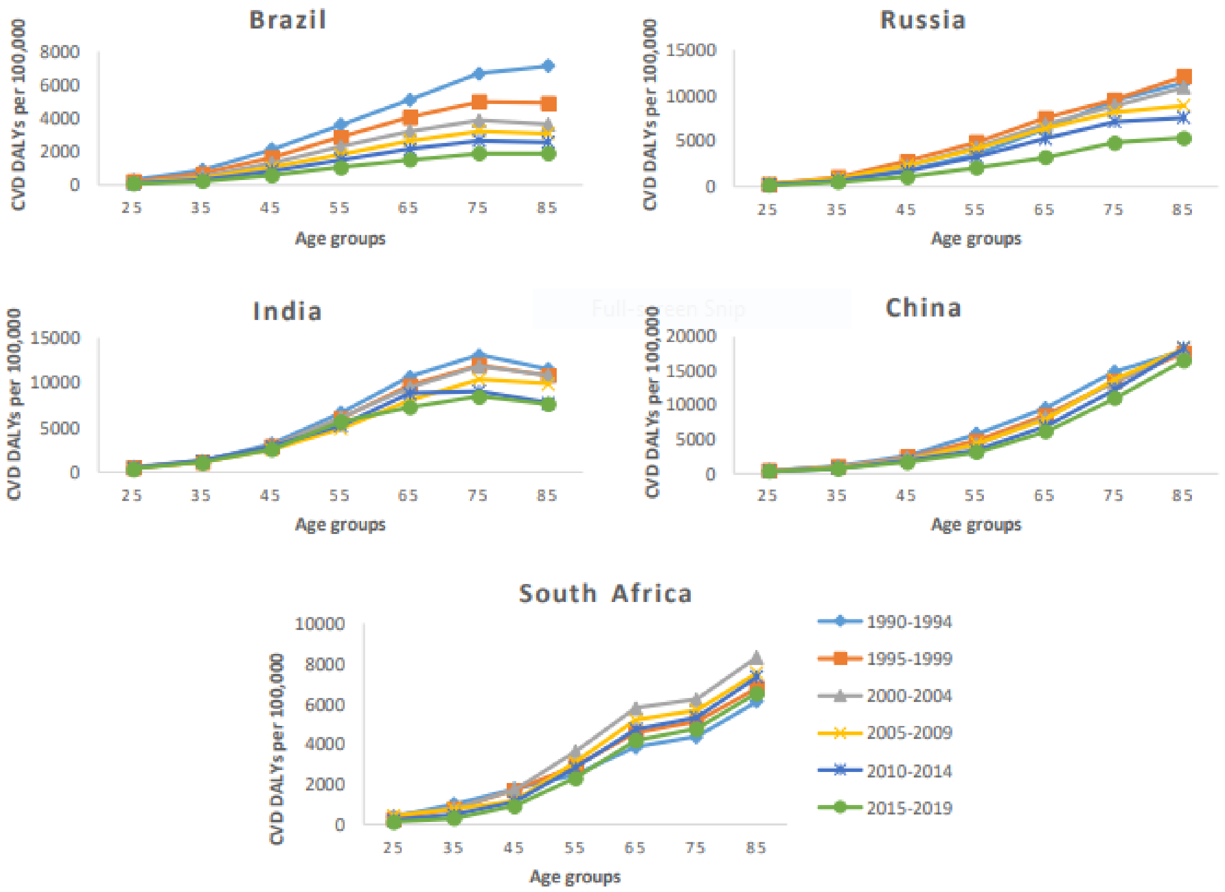

Figure S2: Age-specific disability-adjusted life years (DALYs) rate due to cardiovascular diseases (CVD) attributable to air pollution by period across Brazil, Russia, India, China, and South Africa from 1990 to 2019.

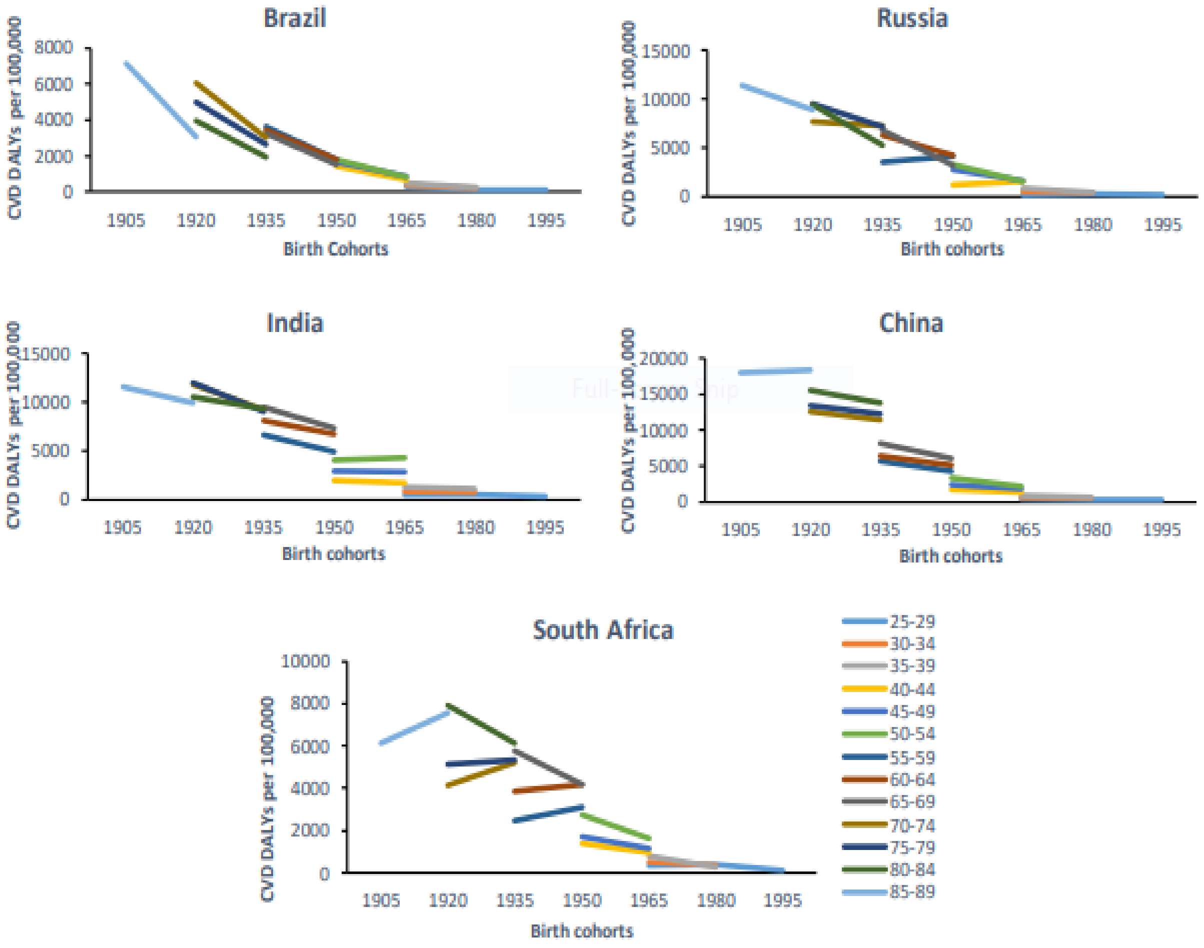

Figure S3: Cohort-specific disability-adjusted life years (DALYs) rate due to cardiovascular diseases (CVD) attributable to air pollution by age groups across Brazil, Russia, India, China, and South Africa from 1990 to 2019.
